# Supplementary material for: Development of a Microfluidic Device for CD4+ T Cell Isolation and Automated Enumeration from Whole Blood
Source: Biosensors (Basel). 2021 Dec 28;12(1):12. doi: 10.3390/bios12010012 (PMC8773767; doi:10.3390/bios12010012)
Supplement: Supplementary file 1 [file biosensors-12-00012-s001.zip › biosensors-1438945-supplementary.pdf]

biosensors

Supplement Materials

Article

Development of a Microfluidic Device for $CD4^+$ T Cell Isolation and Automated Enumeration from Whole Blood

Robert D. Fennell [1,2], Mazhar Sher [1,2] and Waseem Asghar [1,2,3,*]

1 Asghar-Lab, Micro and Nanotechnology in Medicine, College of Engineering and Computer Science, Boca Raton, FL 33431, USA; rfennel1@my.fau.edu (R.D.F.); msher2015@fau.edu (M.S.)
2 Department of Electrical Engineering and Computer Science, Florida Atlantic University, Boca Raton, FL 33431, USA
3 Department of Biological Sciences (Courtesy Appointment), Florida Atlantic University, Boca Raton, FL 33431, USA
* Correspondence: wasghar@fau.edu

I. *Microfluidic Chip Module Design and Construction*

The modules were designed on Autocad. The Autocad software is linked to a UV laser computer that will then cut out the various components. It works best if a composite design of each layer is used. To build, blank out the layers not needed and use those that are visible. Different laser setting is necessary for the desired PMMA and DSA thickness used.

A. *Process for Microchip Module*

1) Clean the glass slide with DI water.
2) Sonicate in Absolute Alcohol for 30 min.
3) Rinse the glass slide with DI water again and dry it with Kim wipe.
4) To place OH^- groups, place the glass slide in oxygen plasma cleaner for 5 min.
5) Treat glass slide immediately by 250 µL of 4% (v/v) 3 MPS. (In two batches each containing 125 µL of 3MPS). Incubate for 45 min at room temperature. (40 uL of 3MPS is added into 960 µL of Absolute Alcohol)
6) Dip rinses the glass slide with 300 uL of absolute alcohol and dry at 110C for 10 min at hot plate inside the fume hood.
7) (Chips can be stored for longer period, if you do not have time for immediate processing). Prepare slides such as APTES slides can be substituted. Assemble modules with activated slips. Assemble microfluidic devices. (Put DSA on PMMA (3.125 mm thick devices)
8) Acquire Maleimide-Neutravidin and inject 20 µL into each channel. Wait 40 min
9) Wash with Pure DPBS twice with 20 µL.
10) Insert 20 µL of 500 µg/mL AntiCD3. Prepare CD3 50 µg/mL, dilute with 900 DPBS, aliquot 20 vials and store at -4-degree C. Take a vial and incubate 5 µm beads for 12 h.
11) Inject 20 µL of Super buffer for 10 min or regular buffer for 60 min.
12) Wash with pure DPBS 20 µL, 2x.
13) Acquire Fresh tested blood. Add EDTA 8 µL per mL of Blood. Divide into control and test portions as desired. Do not dilute test sample for module more than 2 to1. With DBPS. Use the following ratio: Add 20 µL Dynabeads to 200 µL blood and 200 µL of DPBS. Let mix for 20 min. If desired add 1 or 2 drops of NucBlue for specificity measurements.
14) Inject 40 µL of blood per channel, clean surface and wait 10 min.
15) Wash with DPBS at a rate of 50µL minute for 400 µL.
16) Seal input and output ports. Clean device with 70% alcohol or with DI waster depending on module type. Wipe clean with lens paper.

17) Image on inverted microscope (Nikon Eclipse TE2000-S) and point of care wide field imaging device.
18) If possible, use at least 8X stitched Image, or larger in order to get large area to sample low volume of white blood cells on conventional microscopes.
19) Process Images and count cells.

Date:_____________			Process for Capturing cells with antibodies and microbeads Person:_____________________________										Issue H, CD3 Substrates					module type B13b 25 mm channel			
Steps	1	2	3	4	5	6	7	8	9	10	11	12	13	Prep	14	**control**	15	control	16	17	19-20
	Base Material						ALT. APTS slides		prepare slide Alt	X-PBS	UhCT11 biotin CD3		AASJ780 2AP	EDTA	Dynabeads 1145D	NucBlue					
	Strength									no mentals	100ug at 1mg/ml			.5 M		premixed					
	Dilution										10+ 90PBS			8 uL/mL of blood	1/500	2drops/mL					
**module**	**Clean DI**	**Sonic Bath**	**Clean DI**	**Plasma**	**50/950 uL MPS /100 % Al**	**ip Rinse Al Hot plate 100 C**	**Assemble**	**Maleimide -Neutra**	**Arrayit buffer**	**Wash XPBS**		**3 %BSA for 60 minute Superbuffer 10 min**	**Wash X-PBS**	**Mix with Blood sample on arrival**	**CD4 ubeads or Dynabeads mix with blood 1/2**	**NucBlue add to blood with beads.**	**Blood Inject per channel**	**Alexa Fluor sample**	**Wash x-PBS**	**Seal clean**	**Imaging, count**
**Amount**		**Al 100%**			**250 uL**	**100%**		**30 UL**		**40 uL 2X**	**40 uL**	**20 uL**	**40 ul 2x**		**200UL**	**1 drop**	**40 uL**	**1 to 10 ug/mL**	**250 ul**	**70%**	
**Time**		**15 min**		**5 min**	**immed. 30 min**	**10min**		**40 min**			**60 min**	**10 min**			**20 min**	**20 mi**	**10 min**	**1 hour**			
**Act Amt**																					
**Finish**																					
**Start**																					
**Time**																					
**Flow**																					
**Comment**																					

Supplement Figure S1: Process Flow Data Sheet.

B. Process for Imaging and counting Cells in ImageJ

ImageJ software was used to count cells that were photographed on an inverted microscope (Nikon Eclipse TE2000-S) at 10X.
At high resolution the pixel size is .322 µm. The picture image size = length x pixel size X width x pixel size X depth of the modules yeilds the volume of t the volume of the particular picture.
Then the total count divided by the volume gives cells per µL.

Revised Process for ImageJ, Bright Field:

The objective is to have a standard process for counting bright field Images made on the inverted microscope (Nikon Eclipse TE2000-S)
1) Call ImageJ
2) File > open > (go to file image and open) (record pixel size in x, y, note pixel size, and module depth)
3) Process> enhance contrast [.3%]
4) Process > find edges
5) Perhaps Edit > invert if all black background
6) Optional: Process > noise > despeckle if noise in background
7) Optional: Process> subtract background
8) Image > type > 8bit
9) Image > adjust > Threshold (Otsu) Manual adjust to balance desired cells and get rid of other>apply. (Subroutine B)

10) Analyze particles:

11) check image overlay to see if cells were properly counted. Record count and average size. If not redo process.

Supplement Table S1. Key ImageJ parameters for analyze

	CD4+ T cells	CEM cells	Beads	Dapi/NucBlue
Size (number of pixels)	300–900	300–2300	100–250	200–2300
Circularity	.1–1.00	0.1–1.00	0.1–1.00	0.1–1.00
Display result	Yes	Yes	Yes	Yes
Summarize	Yes	Yes	Yes	Yes
Exclude on edges	yes	Yes	Yes	Yes
Include holes	yes	Yes	Yes	Yes

C. Fluorescent Cell Imager (ZOE, Bio-Rad Laboratories, Hercules, CA)

The Fluorescent Cell Imager (ZOE, Bio-Rad Laboratories, Hercules, CA) is capable of bright field, blue field, yellow field and merged field. The machine does not read out a count, but we were able to measure the pixel size be about .389 um on a side. The images were typically 2592 x 1944 pixels. This gives about a 17.25 X multiple with a depth of .76 µm. Table S1 show the details of volume calculations and the cell density calculations.

D. Subroutine B Dapi or NucBlue

The objective is to have a standard process for counting Dapi or NucBlue Images.

1) Call ImageJ
2) File > open > (go to file image and open) (record pixel size in x, y, note pixel size, and module depth)
3) Process> enhance contrast [.3%]
4) Process > find edges
5) Perhaps Edit > invert if all black background

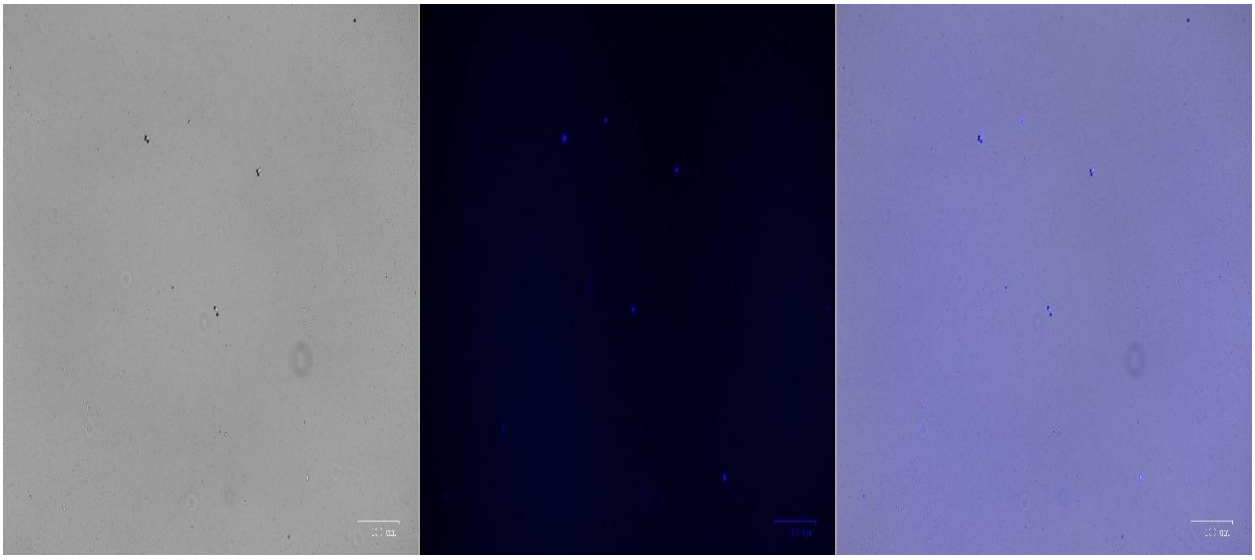

Supplement Figure S2. **From left to right The Bright field Image on** Fluorescent Cell Imager (ZOE, Bio-Rad Laboratories, Hercules, CA)**. The Blue Field Image and The Merge Image, used to calculate Specification. Note the large image but small size of cells**

II *A. Materials and Sources*

**Asghar labs**	**Robert Fennell**	**Oct6 2021**	
**Part name**	**Part number**	**number**	**Internet Location**
NucBlue	R37605	1kit	https://www.thermofisher.com/order/catalog/product/R37605?actionType
anti-CD3 (UCHT1)	ab191112	100 ug	https://www.abcam.com/cd3-antibody-ucht1-biotin-ab191112-references.html
Dynabead CD4	11145D	5 mL	https://www.thermofisher.com/order/catalog/product/11145D?SID=srch-srp-11145D
Strep. Microspheres	24158-1	1mL	http://www.polysciences.com/default/streptavidin-microspheres-6-0-m
Strep. Fluor. Microspheres	24158-1	1mL	http://www.polysciences.com/default/streptavidin-microspheres-6-0-m
EDTA	15575020	4x199mL	https://www.fishersci.com/shop/products/invitrogen-ultrapure-0-5m-edta-ph-8-0/15575020#?keyword=EDTA+invitrogen
DPBS; Alpha Aesar	AAJ7802AP	2 500ml	https://www.fishersci.com/shop/products/phosphate-buffered-saline-dpbs-1x-dulbecco-s-formula-without-calcium-without-magnesium-alfa-aesar/aaj67802ap#?keyword=phosphate+buffer+saline+calcium+and+magnesium+free
Bioton -4-Flourescent	**B10570**	5mg	https://www.thermofisher.com/order/catalog/product/B10570
NeutrAvidin™ Protein, Maleimide-Activated	31007	5m6	https://www.thermofisher.com/order/catalog/product/31007?SID=srch-srp-31007

B. Aliquot for antibodies**.**

Objective: Divide up (Aliquot) antibodies into measurable and usable amounts.
Equipment: Pipette, storage capsules, vented hood, glasses, gloves, storage points.
Material: Anti-CD3 antibody [UCH-T1] (ab22) [1], PBS - phosphate buffered saline.

#	Process	Result	Comment
1	Stock is 50 µg in 100 ml	Receive and store at+ 4-degree C	From ABCAM
2	Dilute with PBS, 900 mL, mix in shaking table. (Time = 20 min.)	Added 900 mL PBS<br>Have 50 µg in 1000 ml	The degree of dilution is dependent on the number of Particles need per experiment.
3	Using pipette divide into 20 vials, use hood, glass and gloves	2.5 µg/50 mL.<br>Store at +4 degree C	1000mL/20 = 50 mL<br>50 µg/20 = 2.5 ug/50mL vial

C. ***Stronger Antibody Binding***

- Antibody affinity refers to the strength of the interaction that the paratope, (the binding site of the antigen) binds to the epitope on the antibody.
- K_A describes how much strength exists at equilibrium.
- $K_{A=\frac{Ab-Ag}{[Ab][Ag]}}$
- K_A = affinity constant
- [Ab] = molar concentration of unoccupied binding sites on the antibody
- [Ag] = molar concentration of unoccupied binding sites on the antigen
- [Ab-Ag] = molar concentration of the antibody-antigen complex
- Antibody Avidity goes further in defining the overall strength and is dependent on affinity, the valency of both antigen and antibody and the arrangement of the antibody versus that of the antigen.
- "Antibody avidity can be improved or careful selection of the antibody is necessary to match the antigen and conditions." Bio-Rad claim 100 to 1000 X improvement.
